# A prospective study on the use of rivastigmine transdermal patch in Alzheimer’s dementia in a routine clinical setting

**DOI:** 10.1590/S1980-57642010DN40300014

**Published:** 2010

**Authors:** Ejaz Nazir, Muhammad Mushtaq

**Affiliations:** 1 Consultant Old Age Psychiatrist, Services For Older People, Shelton Hospital, Shrewsbury, Shropshire, UK; 2 Specialty Registrar, Services For Older People, Shelton Hospital, Shrewsbury, Shropshire, UK.

**Keywords:** rivastigmine, transdermal patch, observational study, longitudinal study, Alzheimer’s dementia, clinical settings

## Abstract

There is not much published literature on the use of rivastigmine patch in a “routine” clinical setting.

**Objectives:**

In this naturalistic longitudinal observational study we sought to evaluate the safety, tolerability and efficacy of the rivastigmine patch in patients with early and late onset moderate Alzheimer’s disease in a routine clinical setting.

**Methods:**

Out of all routine clinical referrals, the first 30 patients with diagnosis of moderate Alzheimer’s dementia who were started on rivastigmine patch were included in the study. Rivastigmine patch dose was titrated from 4.6 to 9.5mg/24 hours as appropriate. The primary outcome measure was safety and tolerability, measured by the incidence of adverse events and discontinuation due to any reason. The secondary outcome measure was to examine improvement on global, functional and behavioral domains as demonstrated by the MMSE (Mini Mental State Examination) score, BADLS (Bristol Activities of Daily Living Skills) score, patient and carer feedback and clinical judgment.

**Results:**

Adverse events were reported in 20% of patients and 10% of total patients needed discontinuation of treatment. Improvement on global, functional and behavioral domains was observed in two thirds of patients whereas one third showed a relative decline. The most common side effect was skin irritation or erythema.

**Conclusions:**

The rivastigmine transdermal patch may provide a treatment option for those patients who require a change in their current oral cholinesterase inhibitor therapy due to safety or tolerability concerns.

## Abstract (pt)

Não há muita publicação na literatura sobre o uso do adesivo de rivastigmina na prática clínica.

**Objetivos:**

Em um estudo observacional longitudinal naturalístico nós tentamos avaliar a segurança, tolerabilidade e eficácia do adesivo transdérmico de rivastigmina em pacientes com doença de Alzheimer moderada de início precoce e tardio.

**Métodos:**

Os primeiros 30 pacientes ambulatoriais com DA moderada de clínicas de referência que iniciaram o uso de adesivo de rivastigmina foram incluídos no estudo. A dose foi escalonada de 4,6 a 9,5mg/24hs quando apropriado. As medidas de desfecho primário foram a segurança e tolerabilidade medidas pela incidência de eventos adversos e descontinuação por alguma razão. A medida de desfecho secundário foi a melhora global, funcional e comportamental, demonstrada pelos escores do Mini-Exame do Estado Mental (MEEM), escores na escala de Atividade de Vida Diária de Bristol, retorno do paciente e cuidador e julgamento clínico.

**Resultados:**

Eventos adversos foram reportados em 20% dos pacientes e 10% deles descontinuaram o tratamento. Melhora em domínios global, funcional e comportamental foi observada em dois terços dos pacientes, enquanto que, no terço restante um declínio relativo foi observado. O efeito colateral mais comum foi irritação ou eritema de pele.

**Conclusões:**

O adesivo transdérmico de rivastigmina pode ser uma opção terapêutica para aqueles pacientes que requeiram mudança na sua terapia oral com inibidor da colinesterase devido à sua segurança e tolerabilidade.

The rivastigmine transdermal patch is the first transdermal treatment for moderate Alzheimer’s disease (AD) and dementia associated with Parkinson’s disease.^1,2^ The efficacy, safety and tolerability of the rivastigmine patch were demonstrated in a large, double blinded, placebo controlled trial that included more than 1000 Alzheimer disease patients. The study gave no indication that patch use may interfere with normal daily activities.^3^ To our knowledge, there is not much published literature on the use of rivastigmine patch in a “routine” clinical setting.

In this naturalistic longitudinal observational study we sought to evaluate the safety, tolerability and efficacy of the rivastigmine patch in patients with early and late onset Alzheimer’s disease in a routine clinical setting. This study was conducted in the Department of Old Age Psychiatry at Shelton Hospital, Shrewsbury, UK over an 18-month period from May 2008-onwards.

## Methods

### Patient selection

As the study planned to assess the rivastigmine patch in a “routine clinical setting”, we selected our patients from routine clinical referrals received to our service for assessment of dementia.

Patients meeting inclusion criteria for the study were women or men aged 50-85 years with a diagnosis of moderate dementia of Alzheimer’s type. Diagnosis was made according to the ICD 10 (International Classification of Diseases, 10th edition).^4^ Each patient underwent a comprehensive evaluation including a neurological examination and appropriate routine laboratory blood tests at baseline. Majority of patients also had a brain scan (Computed Tomography or Magnetic Resonance Imaging or Single Photon Emission Computerized Tomography) done as part of the diagnostic process. Patients included in the study had diagnosis of moderate Alzheimer’s dementia as laid down by NICE (National Institute of Clinical excellence) guidelines.^5^ All patients were living with someone in the community or had a daily contact with a responsible care giver.

Exclusion criteria included mild AD, advanced AD or unstable disease of any type that could interfere with study assessment or with use of the patch or which could pose added risks to patients. We also excluded any other treatable or non treatable conditions other than AD that could explain the dementia.

### Study design

Using a naturalistic longitudinal observational design, we collected and analysed data of the first 30 patients with a diagnosis of moderate AD who were started on rivastigmine patch in our service.

Patients/carers were either interviewed in the clinic or during home visits. We undertook review of patients at baseline and then again at intervals of 3 months and 6 months from baseline. Each review consisted of making an assessment of improvement on global, functional and behavioral domains as demonstrated by the MMSE (Mini Mental State Examination) score, BADLS (Bristol Activities of Daily Living Skills) score, patient and carer feedback and clinical judgment (5). For this study, we aimed to undertake at least 2 assessments for each patient.

The patch was applied by a caregiver to clean, dry, hairless area of skin on the patient’s upper back every morning and worn for 24 hours. Patch placement was altered daily on the back to minimize possible skin irritation. During this period, no restrictions were placed on patient’s normal activities including bathing.

All patients were started on an initial rivastigmine patch dose of 4.6mg/24 hours (patch size: 5cm^2^) and if required doses were titrated to a maximum of 9.5mg/24 hours (patch size: 10cm^2^). More than three quarters (77%) of the patients needed a higher dose.

### Outcome measures

Our primary outcome measure was safety and tolerability measured by the incidence of adverse events and discontinuation due to any reason. As a secondary outcome measure, we examined improvement on global, functional and behavioral domains as demonstrated by the MMSE score, BADLS score, patient and carer feedback and clinical judgment. We did not use the CGI (Clinical Global Impression) scale.

### Ethical considerations

The local research ethics committee waived the need for formal ethical approval. Patients were included in the study after obtaining patients’ and/or carers’ informed consent.

## Results

Being a longitudinal study, patients entered the study at different times within the 18-month study period. At the time of reporting these findings, we had baseline and 3 monthly assessments available for all patients, whereas, 6 monthly scores were available in 24(80%) patients. (Table 1).

**Table 1:** Reported adverse events and availability of efficacy scores at baseline, Month 3 and Month 6.

Criteria	Yes n (%)	No n (%)	Inference
Adverse events reported	6 (20)	24 (80)	diarrhoea: 1, low mood: 1, hallucination: 1, application site skin reactions: 3
Patch discontinued for any reason	3 (10)	27 (90)	2 for rash, one for diarrhea
Baseline MMSE score available	30 (100)	0	
3 monthly MMSE score available	30 (100)	0	
6 monthly MMSE score available	**24 (80)**	**6 (20)**	3 patients discontinued, 1 patient died, 1 patient deteriorated and met exclusion criteria (severe AD), 1 patient had data lost.
Baseline BADLS available	15 (50)	15 (50)	
3 monthly BADLS available	16 (53)	14 (47)	
6 monthly BADLS available	9 (30)	21 (70)	

**Figure 1:**
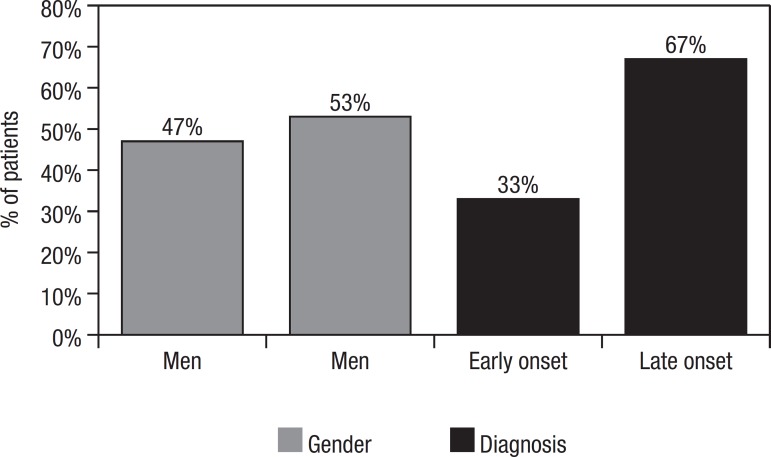
Gender and age distribution of sample.

Our sample comprised 10(33%) cases of early onset AD (median age: 59.5, range 52-64 yrs) and 20(67%) of late onset AD (median age: 79, range 65-88 yrs). Among the participants, 16(53%) were women and 14(47%) men. A total of 29 patients were newly diagnosed whereas one patient was a changeover from donepezil (Figure 1).

Regarding the primary outcome measure in our sample, adverse events were reported in 6 (20%) of the patients. These adverse events included diarrhoea, low mood, hallucination and skin reaction. Overall, 3 (10%) patients needed discontinuation of treatment, where 2 of these discontinuations were due to skin reaction (Table 1).

**Figure 2:**
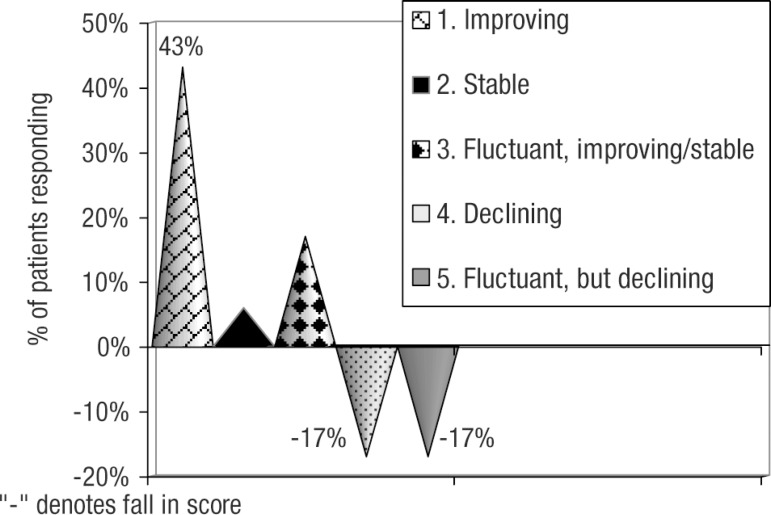
Outcome based on MMSE score.

Regarding the secondary outcome measure, improvements on global, functional and behavioral domains (as demonstrated by MMSE score, BADLS score, patient and carer feedback and clinical judgment) were observed in 20(66%) patients whereas 10(34%) patients showed a relative decline (Table 2 and Figure 2). This global improvement and decline correlated with MMSE scores.

**Table 2:** Outcome based on MMSE score.

Parameter	n (%)	Total: n (%)
Overall improvement in cognitive functions Score with continued improvement Score remained stable Fluctuant score, where final 6 month score either improved or remained stable	13 (43) ** 2 (6)** **5 (17)**	20 (66%)
Overall decline in cognitive functioning Score with continued decline Fluctuant score, where final 6 month score declined	5 (17) 5 (17)	10 (34%)

Review of MMSE score identified 3 trends in patients who showed improvement:

[a] 13(43%) patients showed continued improvement in MMSE score;
[b] 2(6%) patients showed stable MMSE score throughout with no decline; and
[c] 5(17%) patients showed variable MMSE scores (fluctuant score) with final score showed either improvement or remained stable over 6 months of treatment (Table 2, Figure 2).

These cases of fluctuant scores had 3 months of MMSE scores which were less than their baseline scores. This means there was a temporary decline in the score at 3 months on the rivastigmine patch but over a period of 6 months there was no fall in MMSE score compared to their baseline score.

Similarly, we identified 2 trends in patients who showed global decline:

[a] 5(17%) patients showed continued decline in their MMSE score; and
[b] 5(17%) showed variable MMSE scores (fluctuant score).

This means their MMSE score showed a temporary improvement on the rivastigmine patch at 3 months, but their MMSE score at 6 months was lower than their baseline score. (Table 2, Figure 2).

In our study sample, mean MMSE scores at baseline was 19.2. Out of those patients who showed improvement, there was a mean MMSE score improvement of 2.6 and 2.8 points from baseline over a period of 3 and 6 months respectively. However, in patients who did not improve, there was a mean MMSE score decline by 2.8 and 3.7 points over the same periods.

## Discussion

Our sample consisted of one third presenile and two thirds senile dementia patients. Although this is a heterogeneous sample, the proportion of presenile cases (33%) in our sample was far greater than the nationally studied prevalence of presenile dementia. This is explained by our specialist “Younger People with Dementia Service” leading to a high rate of referrals for suspected early onset dementia cases from all over the county.

Rivastigmine is a dual inhibitor of Acetylcholinesterase (ACE) and Butyrylcholinesterase (BuCE), the enzymes that co-regulate synaptic levels of acetylcholine in Alzheimer’s disease patients. Formulating rivastigmine into a transdermal delivery system has the potential to provide smooth and steady inhibition of AChE/BuChE over 24 hours.^12^ Transdermal delivery offers reduced peak-trough fluctuations, continuous drug delivery and an improved tolerability profile.^3,12^. Rivastigmine exposure after application of the maintenance patch dose (9.5mg/24 h) was not significantly different from that achieved after administration of the highest capsule dose of 6mg bid.^12^

Additional advantages of the patch include the convenience of once-daily dosing, simple titration, no requirements for the patient to swallow or take the medication with a full meal, and visual reassurance that the medication has been taken.^6,8^

A 24-week randomized clinical trial has already shown that caregivers preferred the rivastigmine patch to the capsule. Caregivers find it easier to follow treatment schedules with patches.^7,8^ In our observation, patient/carers’ choice was usually influenced by side effects, compliance issues, one less oral tablet to have to take, and by any existing gastrointestinal problems.

Transdermal administration of the patch carries with it the risk of typical adverse events not associated with oral medication such as application site skin irritation and sleep disturbances (because of 24-hour steady rate of drug delivery). Sleep disturbance can be more troublesome compared with oral medication.^9^

Our clinical experience suggests that the most common form of skin irritation is erythema caused by removal of the patch, which normally resolves after a short period of time. Rotating the daily application site of the patch could minimize this irritation. A previous trial of rivastigmine patch versus capsule demonstrated good local skin tolerability (2.4 % discontinuation due to skin reaction) and improved gastrointestinal tolerability (nausea and vomiting incidence being three times lower in the rivastigmine patch group).^3^ In our study, discontinuation due to skin reaction was 6.7%, which is higher than the rate reported in the previous study.

With our baseline mean MMSE score of 19.2, our sample met the NICE recommended definition of moderate Alzheimer’s dementia (i.e.; MMSE score 10-20). In those who responded to the rivastigmine patch, there was a mean improvement in MMSE score of 2.6 and 2.8 points from baseline over a period of 3 and 6 months respectively. This is comparable with results of the Exceed study which has shown an improvement in MMSE score with rivastigmine of 2.35 points over a 2-year period.^11^

Rivastigmine transdermal patch may provide a treatment option for those patients who require a change in their current oral ChEI therapy due to either safety or tolerability concerns, or a lack of therapeutic efficacy.^10^

Replicating the above findings, we also concluded from this study that rivastigmine transdermal patch may provide a treatment option for those patients who require a change in their current oral cholinesterase inhibitor therapy due to safety or tolerability concerns.
